# Non-catecholamine vasopressors in the treatment of adult patients with septic shock—evidence from meta-analysis and trial sequential analysis of randomized clinical trials

**DOI:** 10.1186/s40560-020-00500-0

**Published:** 2020-10-31

**Authors:** Lei Zhong, Xiao-Wei Ji, Hai-Li Wang, Guang-Ming Zhao, Qing Zhou, Bo Xie

**Affiliations:** 1 Department of Intensive Care Units, Huzhou Central Hospital, Affiliated Central Hospital, HuZhou University, 198 Hongqi Rd, Huzhou, 313000 Zhejiang PR China; 2 Department of Obstetrics and Gynecology, Huzhou Central Hospital, Affiliated Central Hospital, Huzhou University, Huzhou, 313000 Zhejiang PR China; 3 Department of Intensive Care Unit, Affiliated Hospital of Yangzhou University, Yangzhou, 225000 Jiangsu Province PR China

**Keywords:** Norepinephrine, Vasopressin, Pituitrin, Terlipressin, Selepressin, Angiotensin II, Septic shock

## Abstract

**Background:**

Norepinephrine (NE) has currently been the first-choice vasopressor in treating septic shock despite generally insufficient for patients with refractory septic shock. The aim of this update meta-analysis was to assess the safety and efficacy of a combination of non-catecholamine vasopressors (vasopressin/pituitrin/terlipressin/selepressin/angiotensin II) and NE versus NE in managing adult septic shock patients.

**Methods:**

We conducted this study of literatures published from the inception to April 30, 2020, using PubMed, Embase, and the Cochrane Library databases without language restriction. Randomized controlled trials comparing NE with non-catecholamine vasopressors among adult septic shock patients were included in this meta-analysis. Pooled effects of relative risk (RR) or standard mean difference (SMD) and corresponding 95% confidence interval (CI) were calculated using a random-effects model.

**Results:**

Twenty-three studies covering 4380 participants were finally enrolled. The combined analysis of non-catecholamine vasopressors resulted in a nonsignificant reduction in 90-day/ICU/hospital mortality except for a decreased in 28-day mortality (*n* = 4217; RR, 0.92; 95% CI 0.86–0.99; *P* = 0.02). This favorable result was subsequently verified by the subgroup analyses of low risk of bias studies (RR = 0.91, 95% CI = 0.84 to 0.98; *P* = 0.02) and catecholamine-resistant refractory shock patients group (RR, 0.84; 95% CI = 0.70–1.00; *P* = 0.048). The pooled analysis of non-catecholamine vasopressors showed a 14% higher success rate of shock reversal at 6 h, a 29% decreased risk of continuous renal replacement therapy, but a 51% increased risk of hyponatremia and a 2.43 times higher risk of digital ischemia. Besides, the pooled data showed that non-catecholamine vasopressors decreased heart rate (HR) (SMD, − 0.43; 95% CI − 0.66 – − 0.19; *P* < 0.001), serum creatinine (− 0.15; 95% CI − 0.29 – − 0.01; *P* = 0.04), and the length of mechanical ventilation (MV) (− 0.19; 95% CI − 0.31 – − 0.07; *P* < 0.01, but there was no significant difference in other parameters.

**Conclusions:**

Current pooled results suggest that the addition of NE to non-catecholamine vasopressors was associated with a marginally significant reduction in 28-day mortality. Moreover, they were able to shorten the length of MV, improved renal function, decreased HR, and increased the 6-h shock reversal success rate at the expense of increased the risk of hyponatremia and digital ischemia.

## Background

Septic shock, as a subset of sepsis, is a phenomenon of persisting hypotension in need of vasopressors to maintain a proper mean arterial pressure (MAP) target ≥ 65 mmHg and having a serum lactate > 2 mmol/L despite aggressive fluid resuscitation [1]. Recent epidemiological data from Europe and North America show the incidence of adult septic shock is around 10.4% at intensive care unit (ICU) admission and at 8.3% at any time during ICU hospitalization, with overall mortality estimated at approximately 38% [2]. To increase the survival of patients with septic shock, appropriate and timely treatment, such as adequate volume, application of antibiotics, followed by the use of catecholamine was utmost important.

Conventionally, norepinephrine (NE), as a potent catecholamine vasopressor, has been a first-line agent to treat septic shock with a target MAP ≥ 65 mmHg recommended by the 2016 Surviving Sepsis Campaign (SSC) Guidelines [3]. Nevertheless, achieving the target MAP may generally require high doses of NE, increasing the risk of myocardial, mesenteric, and digital ischemia as well as arrhythmias and mortality, etc. [4, 5]. Regrettably, around 7% critically ill patients tend to be unresponsive to the increasing dose of NE (refractory shock) in spite of an early recognition, diagnosis, and treatment, with over 50% short-term mortality [6]. Administration of non-catecholamine vasopressors (vasopressin/pituitrin/terlipressin/selepressin/angiotensin II) as alternative or accessory might be beneficial for septic shock patients, especially for patients suffering from catecholamine-resistant refractory shock.

Vasopressin (VP) is a non-specific V1, V2, and V3 receptors agonist that belongs to an endogenous peptide hormone [7]. The rationale for using VP is the relative/absolute insufficiency in septic shock and the vasoconstriction effect mainly through V1 receptor in the vascular smooth muscle, thus restoring vascular tone, achieving a pre-determined MAP goal, and reducing the requirements of catecholamines [8, 9]. Terlipressin (TP) is a V1-selective synthetic analog of VP, thereby decreasing the need of NE and meanwhile avoiding the side effects of V2 activation partly [10]. Selepressin, a novel pure VP V1a agonist, may benefit patients with septic shock in the coming years due to the lack of V2 activity [11]. Angiotensin II (AT-II), as an octapeptide hormone in the renin–angiotensin–aldosterone system, is a powerful vasopressor that induces vasoconstriction by activation of the AT-II type 1 receptor [12].

In the past dozen years since the first randomized controlled trial (RCT) of non-catecholamine vasopressors for the treatment of septic shock was published, there has been a few favorable findings in this field [13–15]. By contrast, these promising results were not validated by other studies [16–28]. Considering these conflicting results, we therefore conducted this study to evaluate whether there is an association between non-catecholamine vasopressors in combination with NE and survival benefit among adult patients experiencing septic shock.

## Methods

Our meta-analysis is reported based on the preferred reporting items for systematic reviews and meta-analyses (PRISMA) guidelines (Additional file 1) [29]. We used the PICO framework for the purpose of defining the clinical question apparently (Additional file 2).

### Search strategy and selection criteria

Two authors (ZL and JXW) independently searched the PubMed, Embase, and Cochrane Library databases from the inception to April 30, 2020, without language restriction, for RCTs that compared the use of non-catecholamine vasopressors vs. NE and evaluated the mortality in adult septic shock patients. The full-search terms and search strategy were available in Additional file 2. The potentially relevant bibliographies of studies were also hand-searched. The exclusion criteria were as follows: age < 16 years, case reports, letters, comments, duplicate publications, reviews, case-control studies, and cohort studies or animal studies. Trial eligibility was done by two authors (ZL and JXW) independently.

### Data extraction and outcome measures

The two authors, independently and in duplicate, extracted data, which was checked by the other authors. The extracted data is as follows (see Table 1). During the process of literature screening, there were 11 different time-point mortalities (6/24 h/3/7/14/28/30/90/180-day/hospital/ICU mortality). The mortality at 30 days was deemed equivalent to 28-day mortality. We ultimately conducted an analysis of 4 different time-point mortalities (28/90-day, ICU, hospital mortality), which is clinically important.

**Table 1:** Baseline characteristics of the included studies

Group	Study ID	Country origin	No. of non-catecholamine vasopressors/NE	Age (years)	Female (%)	APACHE II score	Intervention (non-catecholamine vasopressors)	Comparison (NE)	Center	Setting	MAP target (mmHg)
VP	Dunser et al. 2003 [30]	Austria	8/7	…	...	...	4.00 U/h	MD 2.26 μg/kg/min	S	ICU	≥ 70
	Lauzier et al. 2006 [20]	Multi-country	13/10	54.20	39.13	23.10	0.04–0.20 U/min	0.10–2.80 μg/kg/min	M	ICU	> 70
	Russell et al. 2008 [17]	Multi-country	397/382	60.53	39.02	27.05	ID 0.01 U/min MD 0.03 U/min	ID 5.00 μg/min MD 15.00 μg/min	M	ICU	65–75
	Morelli et al. 2009 [26]	Italy	15/15	65.00	26.67	59.00^a^	FD 0.03 U/min	FD 15.00 μg/min	S	ICU	65–75
	Fonseca-Ruiz et al. 2013 [21]	Colombia	14/16	57.33	40.00	19.20	0.01–0.04 U /min	...	M	ICU	≥ 65
	Oliveira et al. 2014 [13]	Brazil	191/196	...	...	...	0.01–0.03 U/min	0.05–2.00 μg/kg/min	...	ICU	...
	Barzegar et al. 2016 [22]	Iran	15/15	64.00	36.67	...	FD 0.03 U/min	...	S	ICU	≥ 65
	Gordon et al. 2016 [18]	England	205/204	66.51	41.81	23.75	MD 0.06 U/min	MD 12.00 μg/min	M	ICU	65–75
	Hammond et al. 2018 [23]	America	41/41	61.00	51.22	25.00	FD 0.04 U/min	ID 5.00 μg/min MD 15.00 μg/min	S	ICU	≥ 65
	Hajjar et al. 2019 [31]	Brazil	125/125	63.00	45.20	7.00^b^	FD 0.01–0.06 U/min	10.00–60.00 μg/min	S	ICU	≥ 65
VP analogues	Albanèse et al. 2005 [24]	France	10/10	65.50	35.00	28.50	BD 1.00 mg	FD 0.30 μg/kg/min	S	ICU	65–75
	Morelli et al. 2008 [25]	Italy	19/20	66.51	30.77	59.49^a^	BD 1.00 mg	FD 0.90 μg/kg/min	S	ICU	65–75
	Morelli et al. 2009 [26]	Italy	15/15	65.50	23.30	60.00^a^	FD 1.30 μg/kg/h	FD 15.00 μg/min	S	ICU	65–75
	Han et al. 2012 [27]	China	66/73	71.84	28.78	27.34	1.00–2.50 U/ h	...	M	ICU	...
	Svoboda et al. 2012 [16]	Czech Republic	13/17	72.83	40.00	18.00^b^	FD 4.00 mg/24 h	...	S	ICU	65–75
	Xiao et al. 2016 [14]	China	15/17	62.47	31.25	...	FD 1.30 μg/kg/h	ID 0.5.00 μg/min/kg MD 2.22 μg/min/kg	S	ICU	65–95
	Choudhury et al. 2017 [32]	India	42/42	47.53	17.86	14.26^b^	ID 1.30–5.20 μg /min	FD 15.00 μg/min	S	LICU	≥ 65
	Chen et al. 2017 [28]	China	31/26	57.22	49.12	22.05	FD 0.01–0.04 U/min	FD > 1.00 μg/min	S	ICU	65–75
	Russell et al. 2017 [11]	Multi-country	29/21	60.10	44.00	10.48^b^	FD 1.25/2.50 ng/kg/min	...	M	ICU	≥ 65
	Prakash et al. 2018 [15]	India	91/93	...	...	12.51^b^	FD 2.00 mg/24 h	7.50-60.00 μg /min	S	ICU	> 65
	Liu et al. 2018 [19]	China	260/266	61.01	37.07	19.09	ID 20.00 μg/h MD 160.00 μg/h	ID 4.00 μg /min MD 30.00 μg/min	M	ICU	65–75
	Laterre et al. 2019 [4]	Multi-country	562/266	66.31	41.18	25.80	1.70/2.50/3.50 ng/kg/min	...	M	ICU	≥ 65
AT-II	Chawla et al. 2014 [33]	America	10/10	62.85	25.00	30.60	ID 20.00 ng/kg/min MD 40.00 ng/kg/min	…	S	ICU	≥ 65
	Khanna et al. 2017 [34]	Multi-country	163/158	64.00	39.25	28.00	ID 20.00 ng//kg/min MD 200.00 ng//kg/min	…	M	ICU	65–75

*ICU* intensive care unit, *BD* bolus dose, *FD* fixed dose, *ID* initial dose, *MD* maximal dose, *MAP* mean arterial pressure, *M* multi-center study, *S* single-center study, *VP* vasopressin, *TP* terlipressin, *AT-II* angiotensin II, *NE* norepinephrine

^a^SAPS II

^b^SOFA score

The primary endpoint is as follows: 28-day mortality; Secondary endpoints: 90 days, ICU and hospital mortality, ICU length of stay (ICULOS), hospital length of stay (HLOS), the duration of continuous renal replacement therapy (CRRT), and mechanical ventilation (MV); 6-h shock reversal success rate and complications (e.g., hyponatremia, digital ischemia, and acute kidney injury); hemodynamic and metabolic parameters (48 h): heart rate (HR), serum creatinine (Scr), cardiac index, central venous pressure, O_2_ transport index, lactate, left ventricular stroke work index, mean arterial pressure, mean pulmonary arterial pressure, oxygenation index, pulmonary artery occlusion pressure, pulmonary vascular resistance index, right atrial pressure, gastric-mucosal arterial carbon dioxide partial pressure difference, PH, right ventricular stroke work index, stroke volume index, systemic vascular resistance index, mixed venous oxygen saturation; and urine output, O_2_ consumption index.

### Assessment of study quality

The quality of the eligible works was evaluated by two authors (ZL and WHL) in compliance with the Cochrane Handbook for systemic reviews of interventions [35].

### Assessment of risk of bias

This study used sensitivity analysis to assess the robustness of the pooled effect. Additionally, publication bias was assessed by using the funnel plot, Begg’s Test, and Egger’s Test [35].

### Statistical analysis

The pooled effects for dichotomous outcomes and continuous outcomes were expressed as relative risk (RR) with 95% confidence interval (CI) and standard mean difference (SMD) with 95%CI, respectively. Since the data of several studies were presented as median and range/interquartile range, we estimated the mean and standard deviation through the formula provided by Wan and colleagues [36]. The heterogeneity of included trials was defined by the *X*^2^ test, *P* values, and the *I*^2^ statistics. Considering the conservative of random-effects models, we used this model for all pooled analysis [37]. We also conducted a trial sequential analysis (TSA) using random-effects model in order to control the risks of random errors due to sparse data and repetitive testing of cumulative data. The data were accomplished using Review Manager Version 5.3.5 (http://tech.cochrane.org/revman/download), TSA 0.9.5.10 beta (http://www.ctu.dk/tsa/downloads.aspx), and Stata 12.0 software (StataCorp, College Station, TX, USA). All statistical tests were analyzed using two-sided *α* level of 0.05.

The Gordon study [38], as a research branch of the included VASST study [17], mainly researched the cardiopulmonary effects of VP compared with NE in septic shock; therefore, we included this study for analysis of the hemodynamic parameters. Moreover, some data of interest in Dunser [30] come from a previously published individual patient data meta-analysis [39].

Additionally, subgroup analyses according to the risk of bias (low-risk vs high-risk), non-catecholamine vasopressors (VP vs. VP analogs vs. AT-II), and shock types (catecholamine-resistant refractory shock vs. septic shock) were produced. Simultaneously, cumulative meta-analysis of eligible studies comparing NE with non-catecholamine vasopressors in reducing 28-day mortality for adult septic shock patients was carried out based on the publication year.

## Results

There was no RCT which meet our PICO because all the eligible articles were investigated the add-on effect of non-catecholamine. Thus, we compared the effect between non-catecholamine vasopressors + catecholamines and NE group.

### Search results and study characteristics

**Fig. 1:**
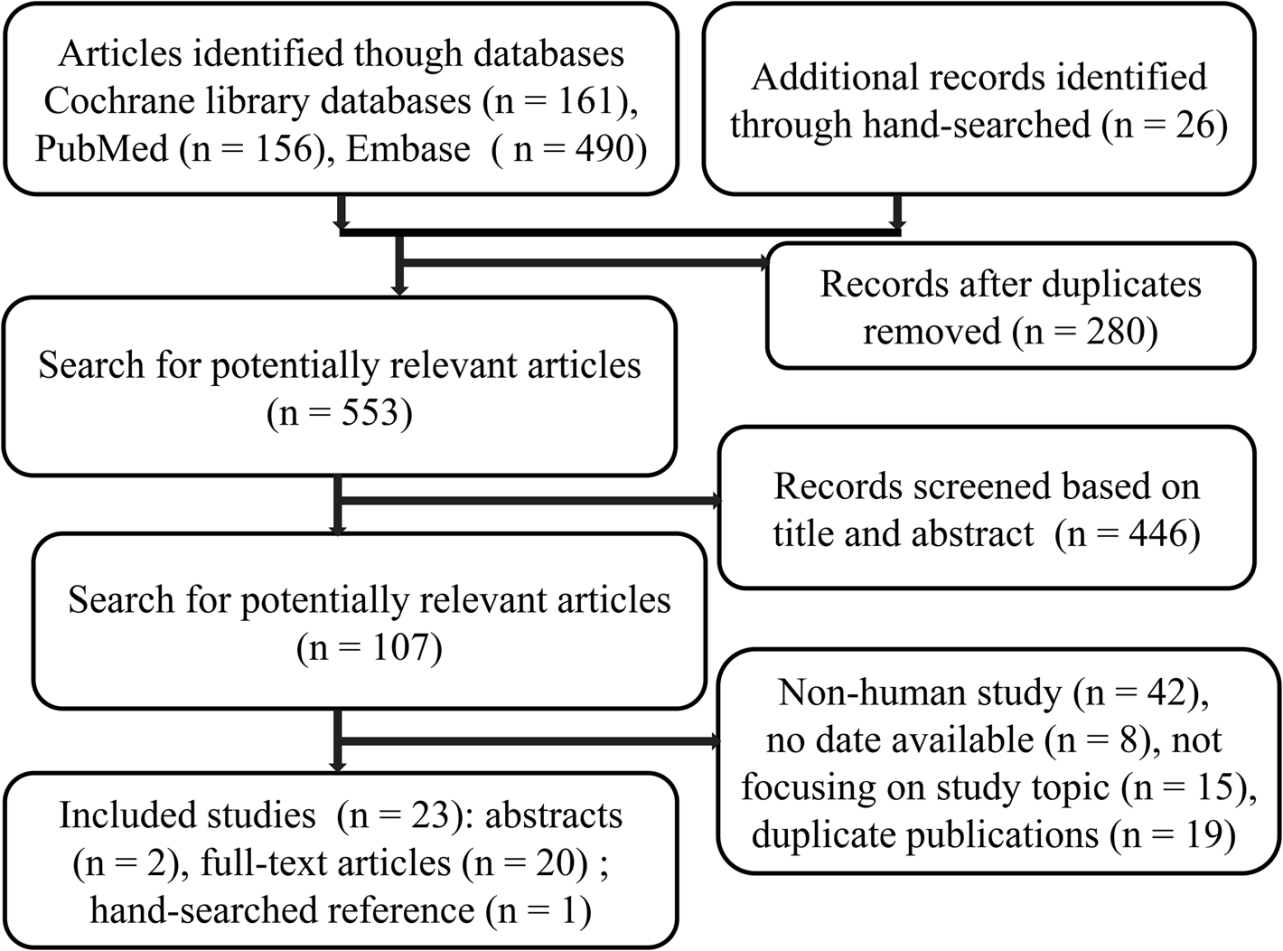
Flow diagram of literature selection procedure

The chart for the study selection process is given in Fig. 1. Up to April 30, 2020, we initially retrieved 807 records and 23 trials reporting on 4380 septic shock patients were eligible for final analysis (21 full-text articles and 2 abstracts). These papers were from Austria [30] (*n* = 1), France [24] (*n* = 1), Iran [22] (*n* = 1), Czech Republic [16] (*n* = 1), England [18] (*n* = 1), Colombia [21] (*n* = 1), Brazil [13, 31] (*n* = 2), India [15, 32] (*n* = 2), Italy [25, 26] (*n* = 2), the USA [23, 33] (*n* = 2), China [14, 19, 27, 28] (*n* = 4), and multi-country [4, 11, 17, 20, 34] (*n* = 5). All these trials from ICUs were published from 2003 to 2019, and the mean age was from 47.53 to 72.83 years. The percentage of female participants ranged from 17.86 to 51.22%, and the mean APACHE II score was between 19.09 and 30.60. The sources of infections are lower respiratory tract (43.1%), abdomen (29.4%), urinary tract (7.8%), skin or soft tissue (3.21%), blood (2.2%), and other (18.4%).

The baseline characteristics of the included trials are exposed in Table 1.

### Assessment of study quality

**Fig. 2:**
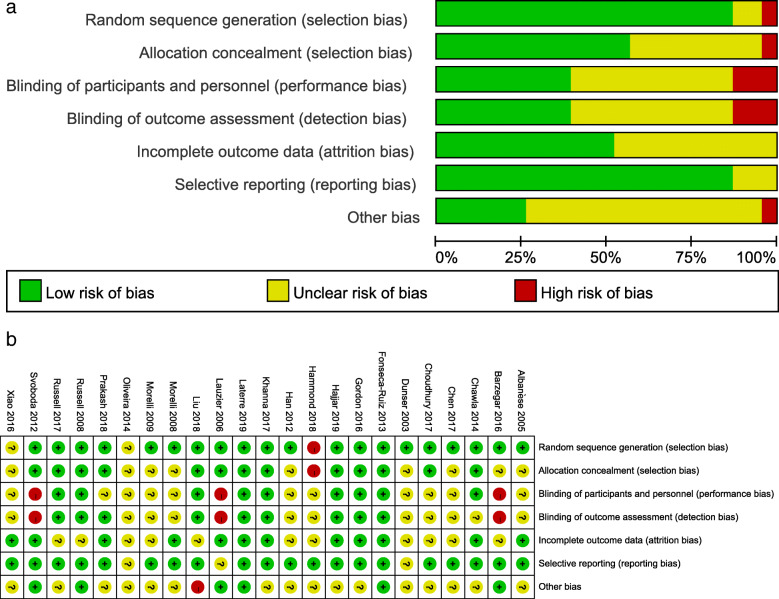
Risk of bias assessment. **a** Risks of bias graph. **b** Risks of bias summary

The risk of bias in the eligible works is depicted in Fig. 2. A high risk of both performance and detection bias was existed in three studies [16, 20, 22] on account of the lack of blinding. There was a high risk of other potential sources of bias in the Liu study [19] because of the early termination of schedule. The Hammond study [23] was subject to selection bias, as the conclusion might be influenced by seasonality effects—the allocation method in this study was on the basis of a predetermined time period instead of the randomization principle.

### Sensitivity analysis and assessment of reporting biases

The sensitivity analysis suggested no single study had a significant influence on the pooled RR, revealing the stability and reliability of the findings (28-day mortality).

**Fig. 3:**
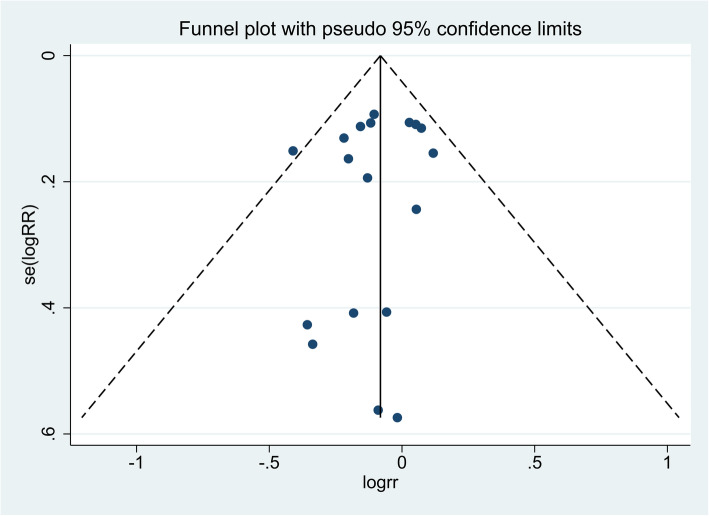
Funnel plot assessing publication bias. The blue dots and dotted line represent individual studies and 95% confidence intervals, respectively

As noted in Fig. 3, no significant publication bias of the primary outcome (28-day mortality) among the included studies was observed via visual inspection of the funnel plot, which was verified by the statistical tests (Begg test, *P* = 1.00; Egger test, *P* = 0.47; Additional file 3).

### Synthesis of primary outcomes

**Fig. 4:**
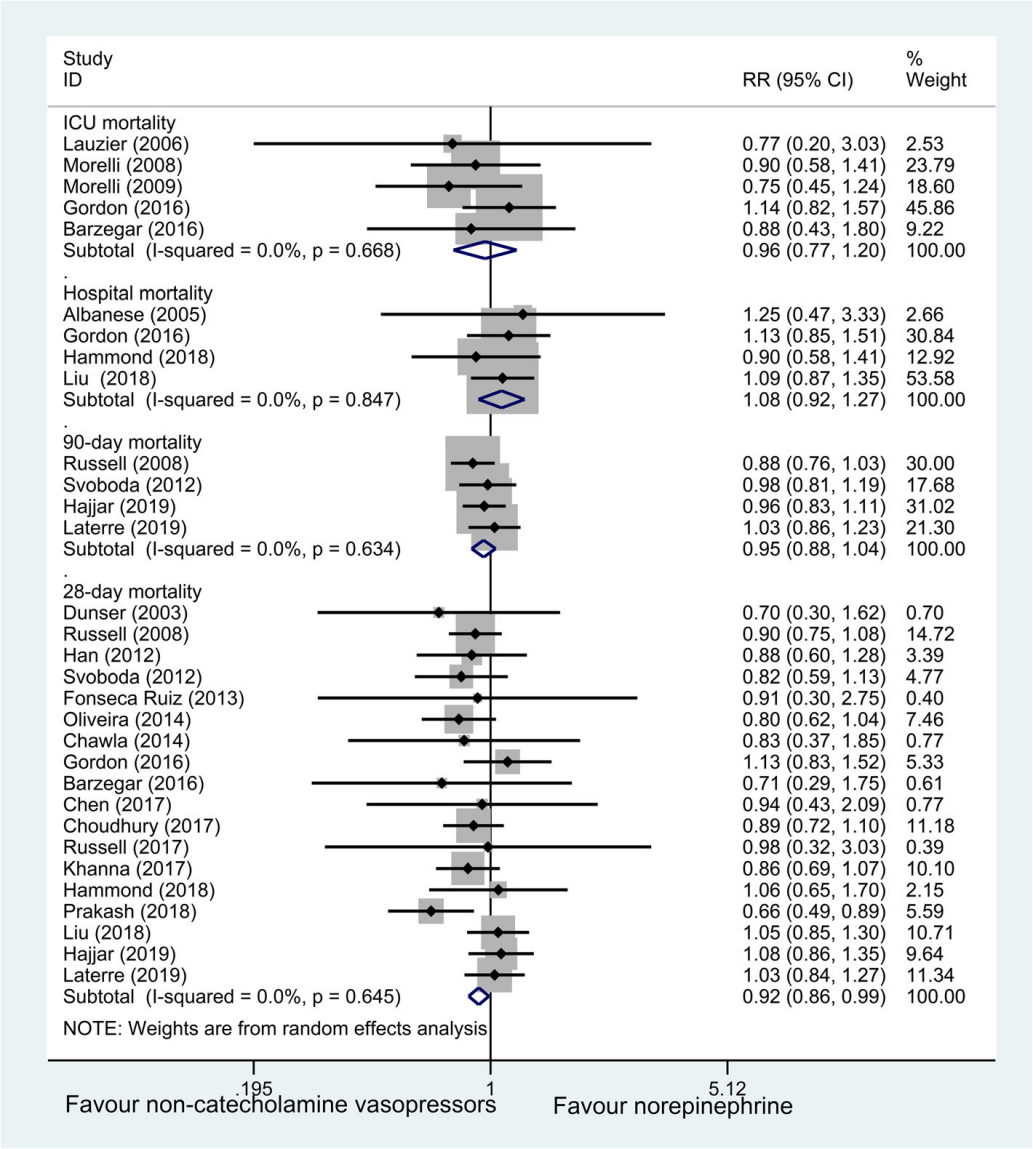
Forest plot for 4 different time-point mortalities of non-catecholamine vasopressors versus NE treatment. NE norepinephrine, ICU intensive care unit, RR relative risk, CI confidence interval

Mortalities at 4 different time-points (28/90-day, ICU, hospital mortality) data were available from 23 trials with 4380 patients. In comparison with NE group, non-catecholamine vasopressors plus NE treatment were associated with a marginally lower 28-day mortality rate (*n* = 4217; RR, 0.92; 95% CI 0.86–0.99; *P* = 0.02), with no obvious heterogeneity among these studies, but this group had no significant effect on 90-day, ICU, and hospital mortality (*P* > 0.05, Fig. 4).

**Fig. 5:**
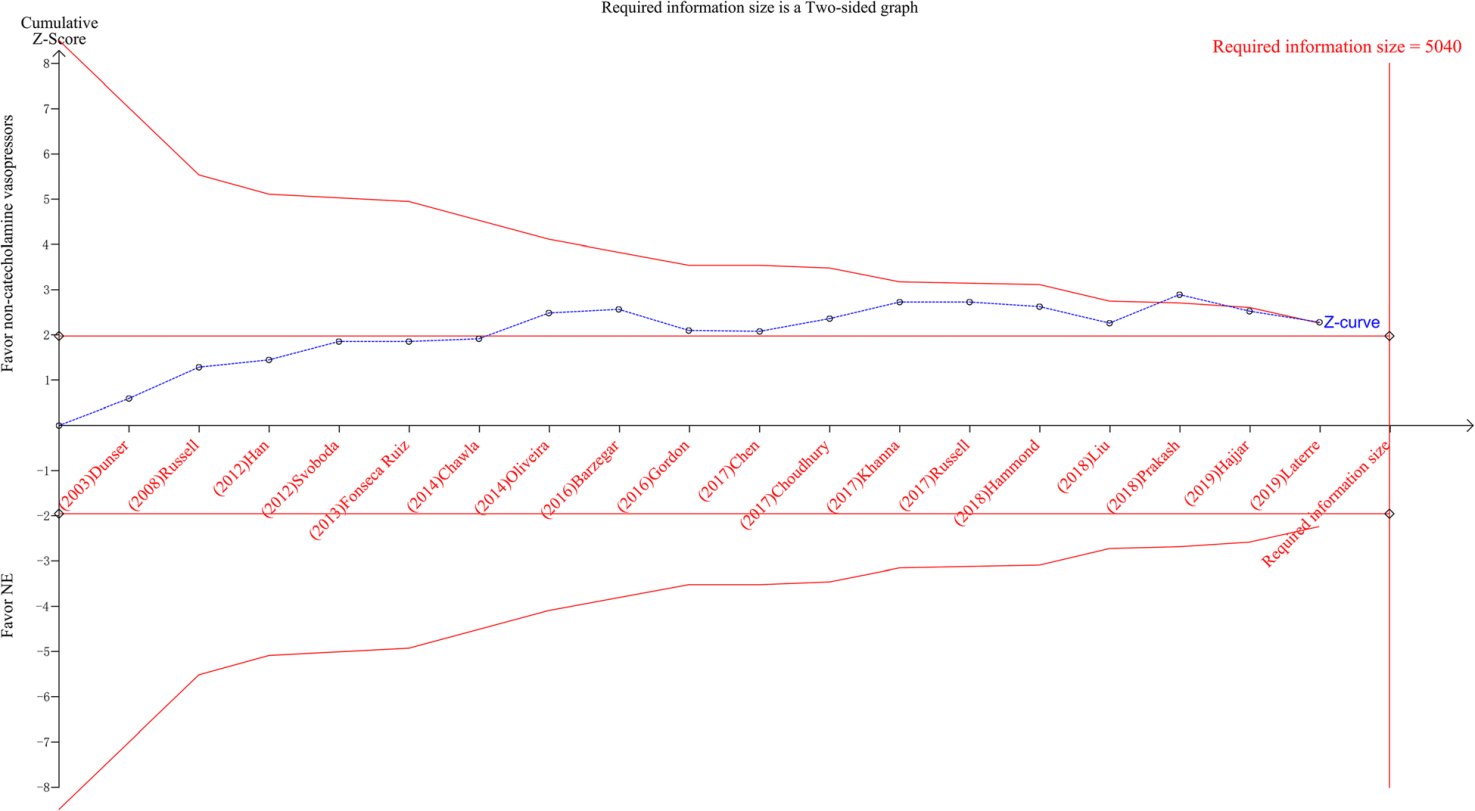
Trial sequential analysis for 28-day mortality. Heterogeneity adjusted required information size of 5040 patients calculated on basis of proportion of 28-day mortality of 38.26, 42.13% in the non-catecholamine vasopressors and norepinephrine group, respectively (*α* = 5%, *β* = 20%, *I*^2^ = 0%)

TSA result of 28-day mortality showed that 4217 (83.67%) of the required information size (RIS) of 5040 patients was accrued. Although the cumulative *Z*-curve (blue line) had surpassed the conventional boundary line obviously and adjusted boundary line favoring the intervention group slightly, it did not reach the optimal information size, indicating a potential possibility of negative result (Fig. 5).

The analysis results of several endpoints are displayed in Table 2.

**Table 2:** The summary results of all 23 studies

Results	Primary outcomes				Subgroup analyses						
	28 days	90 days	ICU	Hospital	Low risk	High risk	VP	VP analogs	AT-II	Refractory shock	Septic shock
RR	0.92	0.95	0.96	1.08	0.91	0.97	0.95	0.91	0.85	0.84	0.94
95% CI lower bound	0.86	0.88	0.77	0.92	0.84	0.83	0.85	0.81	0.69	0.70	0.87
95% CI upper bound	0.99	1.04	1.20	1.27	0.98	1.15	1.06	1.02	1.06	0.998	1.01
*P* value	0.02^*^	0.26	0.73	0.35	0.02^*^	0.75	0.33	0.11	0.14	0.048^*^	0.11

*ICU* intensive care unit, *VP* vasopressin, *AT-II* angiotensin II, *RR* relative risk, *CI* confidence interval, *P < 0.05

### Synthesis of secondary outcomes

#### The duration of hospital, ICU, CRRT, and MV

In all patients concerned, a significant smaller reduction in the length of MV (SMD = − 0.19; 95% CI = − 0.31 to − 0.07; *P* < 0.01) was noted in the non-catecholamine vasopressors group, but not for ICULOS, HLOS, and the duration of CRRT, with different levels of heterogeneity among these studies (Additional file 3).

#### A 6-h shock reversal success rate and the incidence of complications

The pooled analysis showed that the non-catecholamine vasopressor group revealed a significant difference in a 6-h shock reversal success rate (*n* = 330; RR, 1.14; 95% CI = 1.05 to 1.23; *P* < 0.01), CRRT (*n* = 869; RR, 0.71; 95% CI = 0.56 to 0.89; *P* < 0.01), hyponatremia (*n* = 1584; RR, 1.51; 95% CI = 1.04 to 2.20; *P* = 0.03), and digital ischemia (*n* = 3329; RR, 2.43; 95% CI = 1.18 to 5.00; *P* = 0.02) (Additional file 3).

#### Hemodynamic and metabolic parameters (48 h)

Compared with the NE group, the non-catecholamine vasopressors showed a significant reduction in HR (SMD = − 0.43; 95%CI = − 0.66 to − 0.19; *P* < 0.001) and SCr (SMD = − 0.15; 95%CI = − 0.29 to − 0.01; *P* = 0.04), and there was no significant difference on other hemodynamic and metabolic parameters (Additional file 3).

### The results of subgroup analysis

The RR was 0.91 (*n* = 3549, 95% CI = 0.84 to 0.98; *P* = 0.02) for a low risk of bias studies with significant difference and 0.97 (*n* = 668, 95% CI = 0.83 to 1.15; *P* = 0.75) for high-risk of bias studies without statistical significance. In the subgroup analysis stratified by the non-catecholamine vasopressors types, they did not show any significant difference in 28-day mortality, compared with NE group (*P* > 0.05). In the subgroup analysis according to shock types, a marginal improvement in 28-day mortality was discovered in patients with catecholamine-resistant refractory shock (*n* = 396, RR, 0.84; 95% CI = 0.70 to 1.00; *P* = 0.048), whereas the difference in septic shock patients was not significant (Additional file 3).

### Cumulative meta-analysis

The cumulative meta-analysis suggests that a positive result first appeared in 2014 Oliveira [13] (cumulative RR, 0.86; 95% CI = 0.76 to 0.97), and this trend seems relatively stable over time (Additional file 3).

## Discussion

Our study of 23 RCTs involving 4380 individuals compared the safety and efficacy of non-catecholamine vasopressors versus NE in improving survival among patients with septic shock and concluded that the potential vasopressors treatment might be associated with improved 28-day mortality, which was subsequently verified by a cumulative meta-analysis and TSA. However, the favorable result of TSA should be regarded with caution due to an insufficient RIS and could not be confirmed by the majority of subgroup analyses. Significantly, subgroup analysis stratified by shock types was consistent with the main outcome partly and in particular for catecholamine-resistant refractory shock patients, the RR was 0.998. These patients remain a major troublesome clinical problem, which is in relation to the uncontrolled pathologic vasodilation (excessive production of nitric oxide) and vascular hypo-responsiveness to endogenous vasoactive hormones (i.e., cortisol, VP, and AT-II) [6]. Therefore, further study should focus on assessing the vasopressors’ sensitivity to vascular endothelial cell with the purpose of individualized treatment.

Compared with the NE group, patients receiving non-catecholamine vasopressors experienced a significant reduction in the length of MV, and a nonsignificant decrease in the ICULOS, HLOS, and duration of CRRT. Besides, compared to those treated with NE, patients treated with non-catecholamine vasopressors had a 14% higher success rate of shock reversal at 6 h, a 29% decreased risk of CRRT, but a 51% increased risk of hyponatremia and a 2.43-fold higher risk of digital ischemia. When compared to NE, non-catecholamine vasopressors are related to decreased HR and SCr, but had no difference in cardiac index (CI). The potential renoprotective effects of these agents, such as decreasing the concentration of SCr and risk of CRRT, have been confirmed in our study, compared with NE monotherapy.

NE has been the frontline treatment since the SSC guidelines were first published in 2004, but it is still not perfect. This adrenergic agent causes increased oxidative stress and has harmful biological effects on the inflammatory response and cell energy metabolisms [40]. In this respect, the concept of “decatecholaminization,” defining as a partially or completely alternative to catecholamines rather than look for the best catecholamine, emerged in the last decade in fear of catecholamine exposure, resulting in a requirement of non-adrenergic receptors like VP, TP, selepressin, and AT-II [41–43], and then began a fight against mortality between adrenergic and non-adrenergic vasopressor agents.

The overwhelming findings from the majority of studies, including a Cochrane review [44], several trials [23, 28], reviews, and meta-analyses [5, 39, 45], determined that there is nonsignificant difference in mortality between shock patients who received non-adrenergic agents and those who received catecholamines. One early meta-analysis by Zhou and colleagues [45] with seven trials in 2014 acknowledged that there is insufficient evidence to conclude that VP is not inferior to NE in improving 28-day survival rate and hemodynamics, which was further confirmed by a recent individual patient data meta-analysis with four trials in 2019 [39]. In contrast to these unfavorable results, several meta-analyses [46–48] illustrated that the use of non-adrenergic vasopressors is linked with reduced mortality. So for example, a latest meta-analysis in 2019 with twenty studies has demonstrated that as compared with using catecholamines, the application of VP receptor agonists decrease mortality in patients presenting with septic shock in spite of an increased risk of digital ischemia [47].

Not only that, a Cochrane review believed that there is not sufficient evidence to indicate that any of the investigated vasopressors (NE/TP/AVP/epinephrine/dopamine) are superior over others regarding mortality [44]. As mentioned in several reviews [41, 49], the struggle between adrenergic and non-adrenergic vasopressors seem to be internecine; thus, a multimodal strategy with two or more vasopressors may be reasonable. Furthermore, the contemporary SSC guidelines also supported that NE is a first-choice vasopressor, and the addition of either VP or epinephrine for patients who are hypo-responsiveness to vasopressors as second-choice options is desirable [3]. In clinical practice, concomitant NE and VP infusions for septic shock patients are the common phenomena [50]. In view of these, we consider that “decatecholaminization” would be premature before the advent of a powerful alternative vasopressor.

To our knowledge, our study might be the first cumulative meta-analysis to evaluate the efficacy and safety of non-catecholamine vasopressors (vasopressin, pituitrin, terlipressin, selepressin, and angiotensin II) versus NE in managing adult septic shock patients. Prior to this, different time-point mortalities (i.e., 28/30/ICU/hospital mortality) were regarded to be equal for analysis in the majority of previous meta-analyses. Instead, our study was based upon the viewpoint of 4 different time-point mortalities (28/90-day/ICU/hospital mortality), which to some extent reflects a reliable effect of non-catecholamine agents on mortality in patients with septic shock. Our result, in line with some of existing meta-analyses, suggests that the use of non-adrenergic agents might have led to a reduced of 28-day mortality in patients with septic shock, which is further confirmed by the result of cumulative meta-analysis and TSA.

Various potential limitations should be mentioned. First, the plasma levels of VP were not collected in most of included studies; thus, it is difficult to determine whether an absolute or relative deficiency of this endogenous hormone and the actual pharmacological effect of exogenous hormone on septic shock patients. As a guide for the addition of exogenous hormone therapy, the measurement of plasma VP concentrations is needed in future investigation. Second, the maximum doses of the study drugs and open-label NE were infused notwithstanding, patients who still failed to achieve the target MAP were in need of a rescue therapy, that is, more than one other open-label vasopressors (i.e., dopamine, epinephrine, and phenylephrine) was used, bringing about a confounding effect. Moreover, trials that comparing the concomitant use of non-catecholamine agents and NE (open-label NE was additionally infused if target MAP was not maintained) versus NE is also factored into the analysis in our study. Third, the results might be influenced by the different initial times, dosages, and infusion methods of non-catecholamine vasopressor agents among studies. Finally, despite a low level of statistical heterogeneity of our study, we cannot ignore the heterogeneity of clinical and methodological, which is related to the differences of patients, interventions, endpoints, research designs, and qualities. Therefore, further large prospective RCTs, especially for the studies with long-term follow-up (such as 90/180-day mortality), might be needed in this area to verify our results.

## Conclusions

In conclusion, pooled data from 23 trials suggest that concomitant non-catecholamine vasopressors and NE treatment could marginally improve 28-day mortality and are associated with shortened the length of MV, improved renal function, decreased HR, and increased the success rate of the target MAP at 6 h at the price of increased the risk of hyponatremia and digital ischemia.

## Supplementary Information

**Additional file 1:.** The PRISMA checklist

**Additional file 2:.** The PICO framework, search strategy and search results

**Additional file 3:.** Sensitivity analysis, reporting biases and forest plots

## Data Availability

Not applicable.

## Abbreviations

AT-II: Angiotensin II
CRRT: Continuous renal replacement therapy
CI: Confidence interval
HR: Heart rate
HLOS: Hospital length of stay
ICUs: Intensive care units
ICULOS: ICU length of stay
MAP: Mean arterial pressure
MV: Mechanical ventilation
NE: Norepinephrine
RCTs: Randomized controlled trials
RR: Relative risk
Scr: Serum creatinine
SMD: Standard mean difference
TP: Terlipressin
TSA: Trial sequential analysis
VP: Vasopressin
