# Supplementary material for: Non-catecholamine vasopressors in the treatment of adult patients with septic shock—evidence from meta-analysis and trial sequential analysis of randomized clinical trials
Source: J Intensive Care. 2020 Oct 31;8:83. doi: 10.1186/s40560-020-00500-0 (PMC7603734; doi:10.1186/s40560-020-00500-0)
Supplement: Supplementary file 3 — Additional file 3:. Sensitivity analysis, reporting biases and forest plots [file 40560_2020_500_MOESM3_ESM.docx]

**Sensitivity analysis**

**Assessment of reporting biases**

**Begg's Test**

adj. Kendall's Score (P-Q) = -1

Std. Dev. of Score = 26.40

Number of Studies = 18

z = -0.04

Pr > |z| = 0.970

z = 0.00 (continuity corrected)

Pr > |z| = 1.000 (continuity corrected)

**Egger's test**

------------------------------------------------------------------------------

Std_Eff | Coef. Std. Err. t P>|t| [95% Conf. Interval]

-------------+----------------------------------------------------------------

slope | -.0343341 .0715849 -0.48 0.638 -.1860874 .1174191

bias | -.3492758 .4724444 -0.74 0.470 -1.350813 .6522616

------------------------------------------------------------------------------

**Funnel plots**


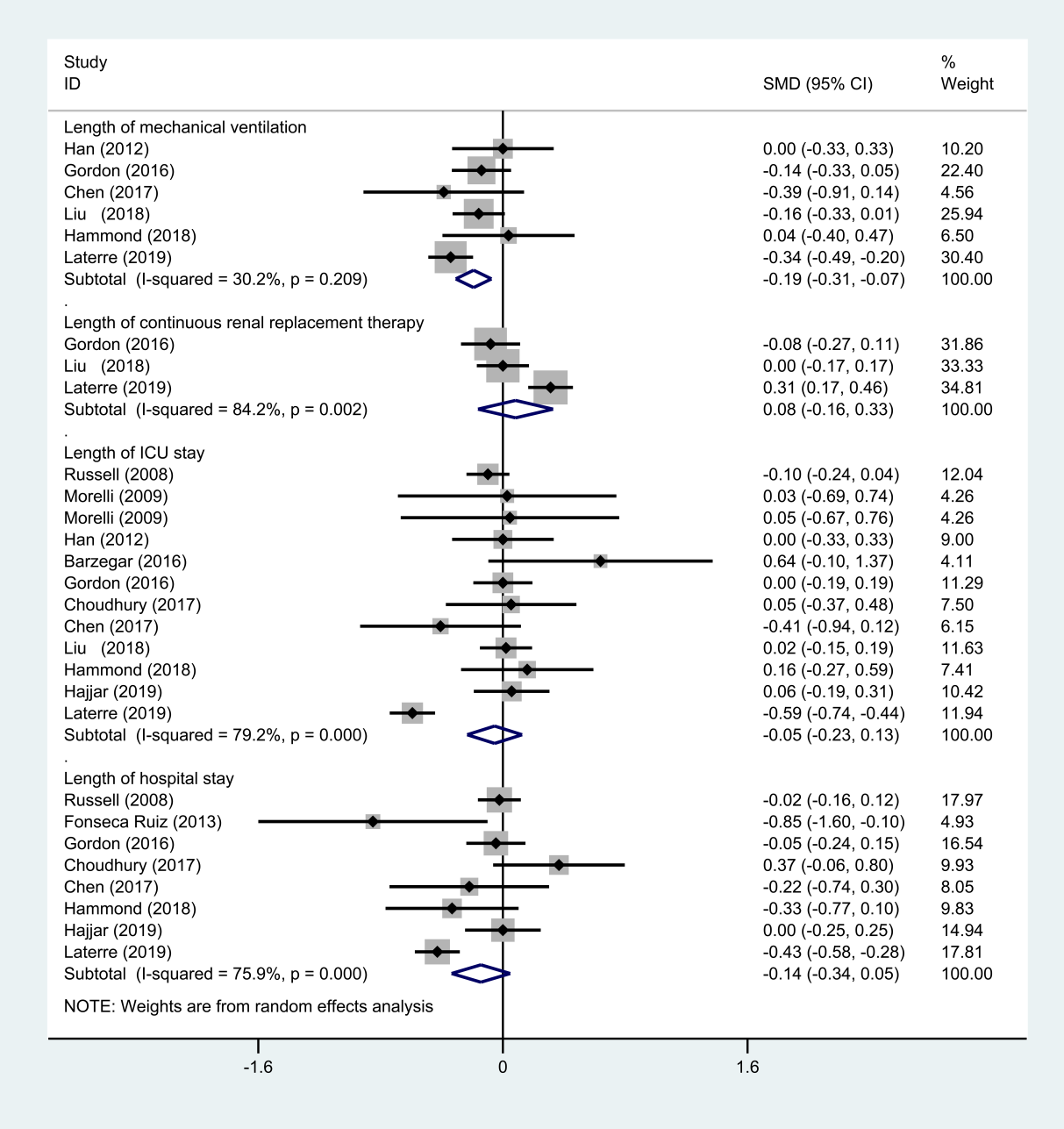


**a.** Forest plot for effects of non-catecholamine vasopressors on the duration of CRRT, MV, ICU and hospital stay. ICU intensive care unit, MV mechanical ventilation, CRRT continuous renal replacement therapy, SMD standard mean difference, CI confidence interval.


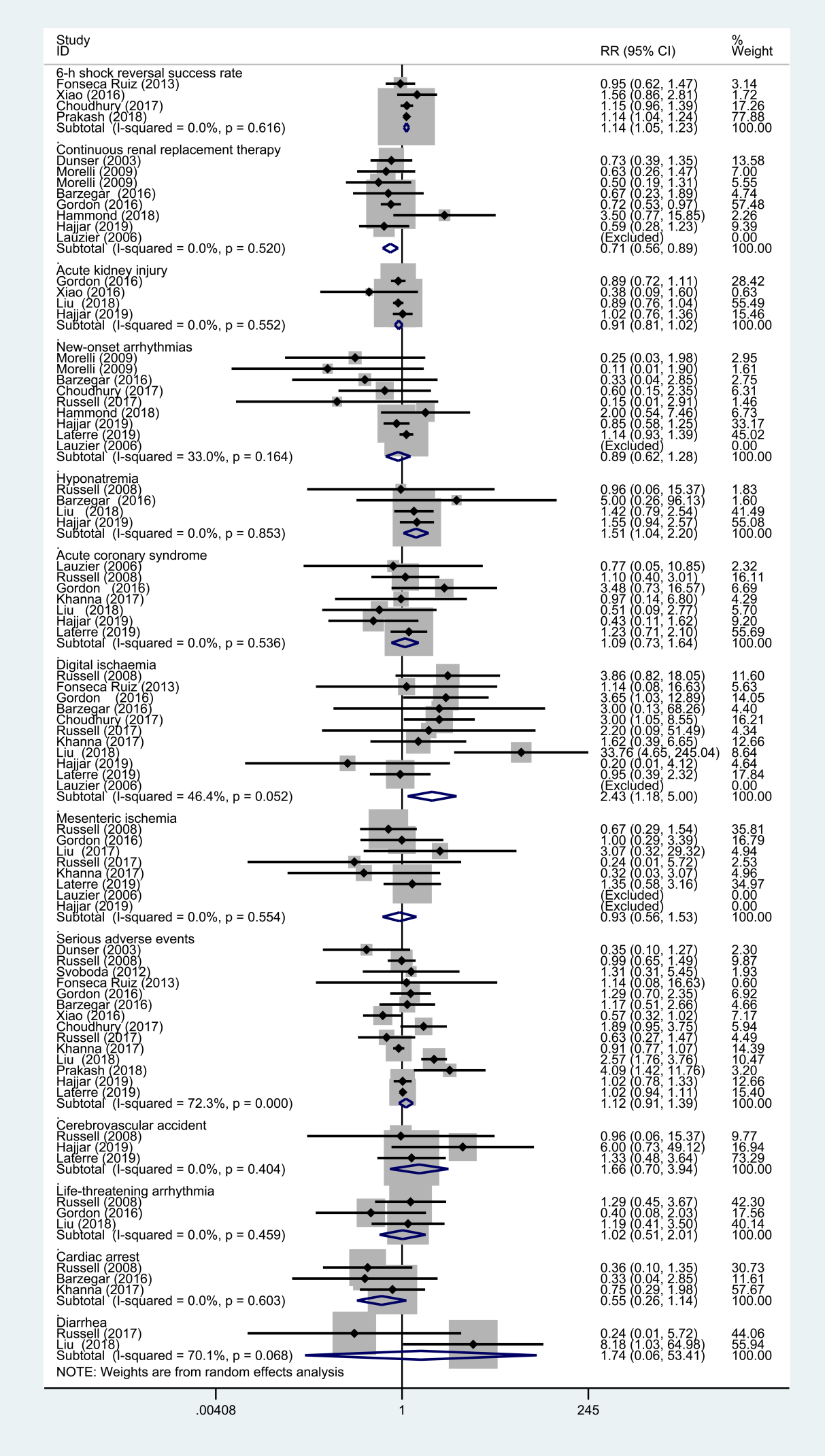


**b.** Forest plot for the 6-h shock reversal success rate and the incidence of complications of non-catecholamine vasopressors versus NE treatment. NE norepinephrine, RR relative risk, CI confidence interval.

**
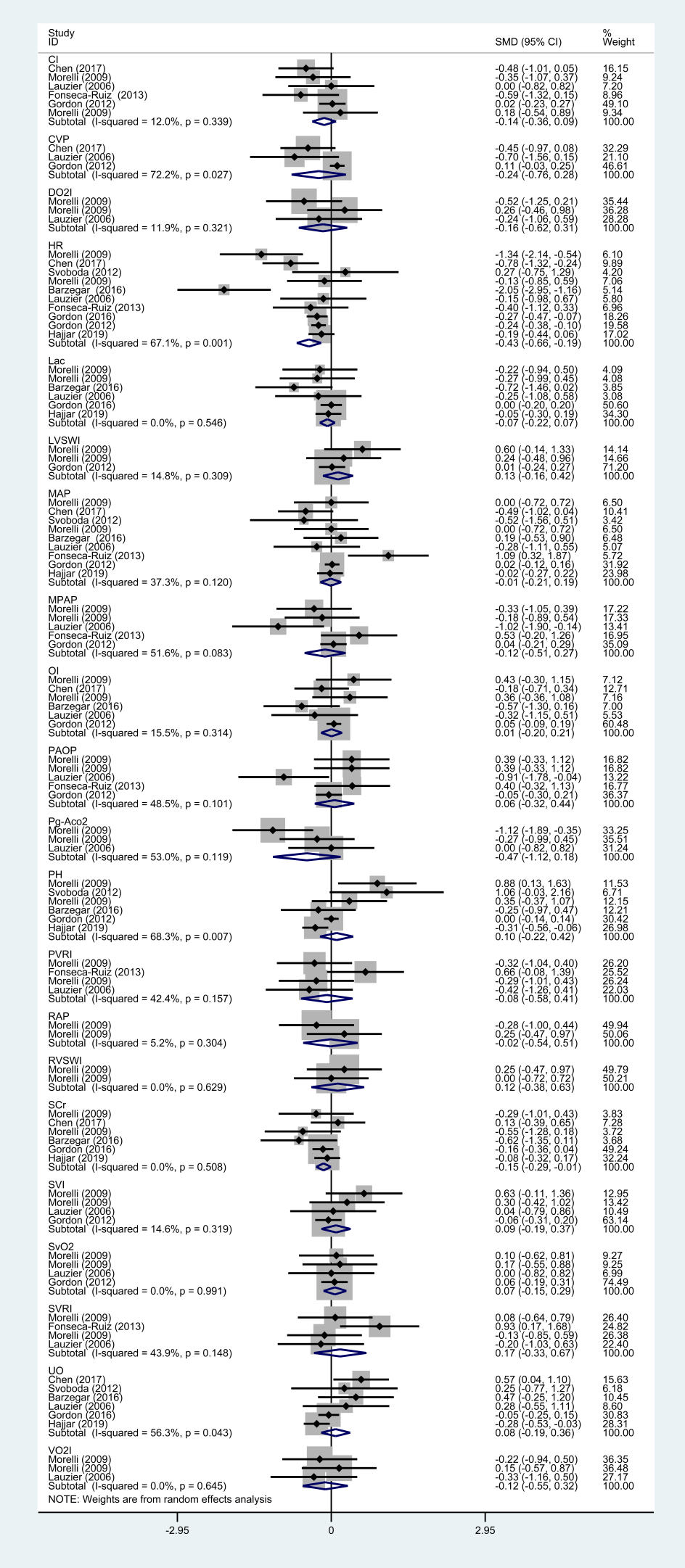
**

**c.** Forest plot of association between use of non-catecholamine vasopressors and the hemodynamic and metabolic parameters. RR relative risk, CI confidence interval.


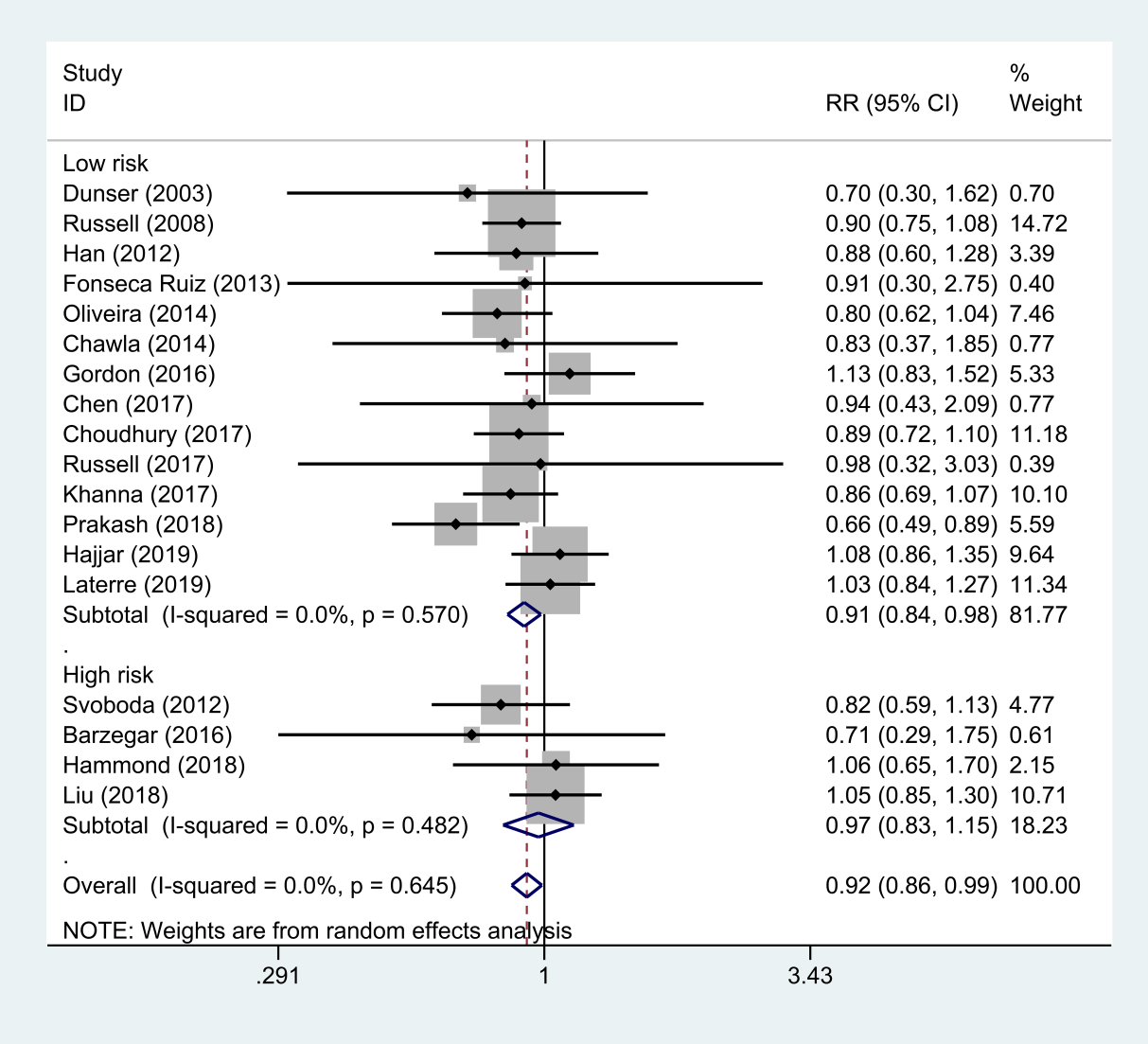


**d．**Forest plot of subgroup analysis by the risk of bias (low risk vs high risk). AT-II angiotensin II, VP vasopressin, RR relative risk, CI confidence interval.


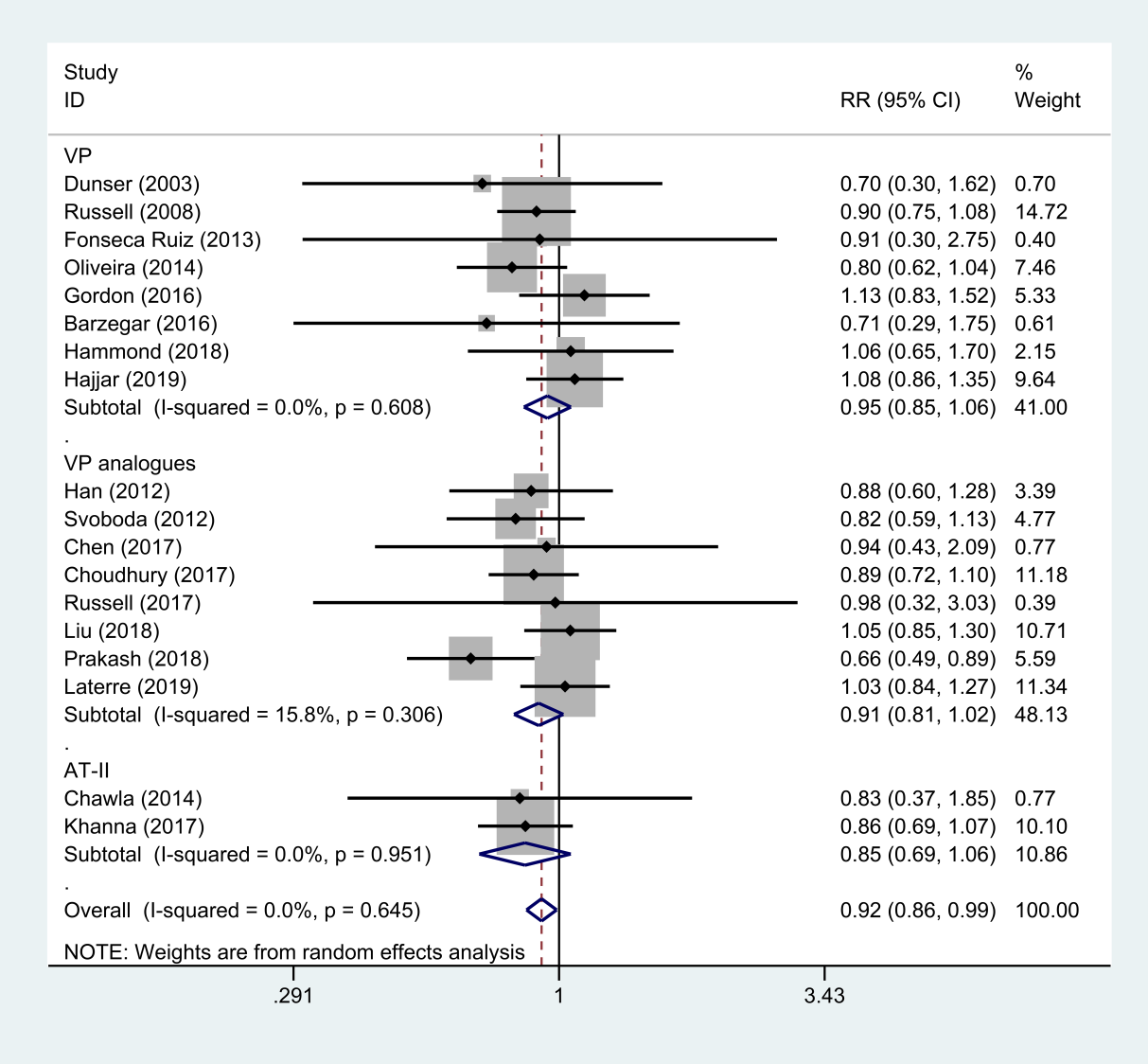


**e.** Forest plot of subgroup analysis by type of non-catecholamine vasopressors (VP vs. VP analogues vs. AT-II). AT-II angiotensin II, VP vasopressin, RR relative risk, CI confidence interval.


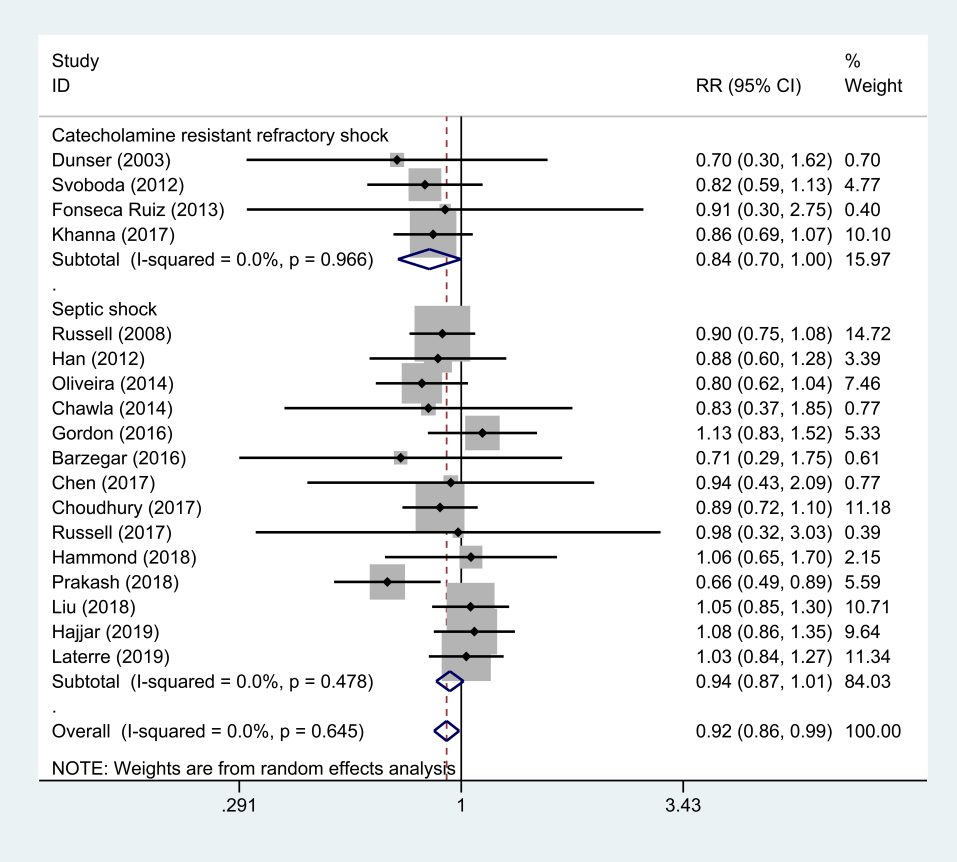


**f.** Forest plot of subgroup analysis by the type of shock. RR relative risk, CI confidence interval.


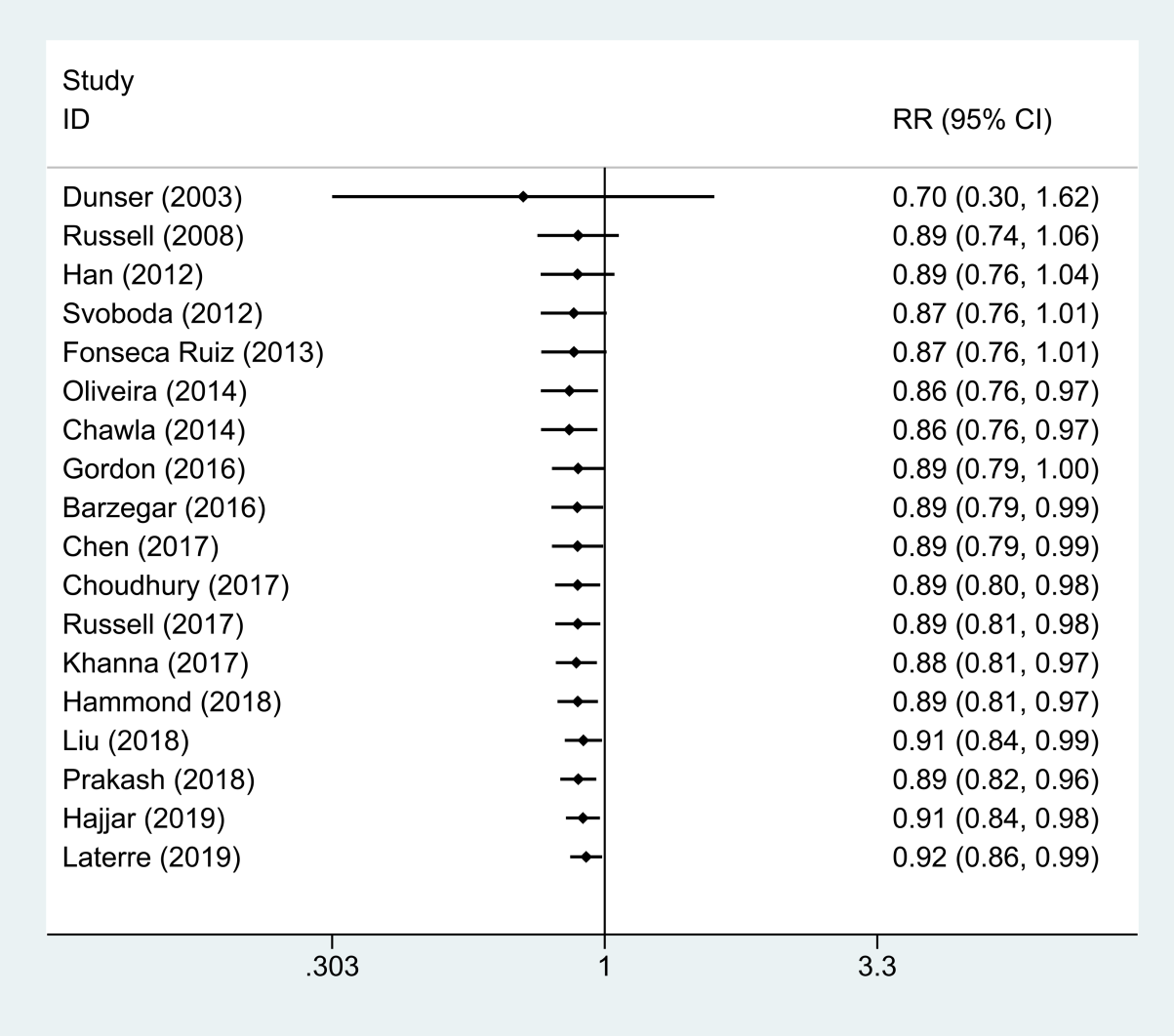


**g.** Forest plot for cumulative meta-analyses comparing non-catecholamine vasopressors with NE in 28-day mortality. RR relative risk, CI confidence interval.

**Note：**

AT-II: angiotensin II, CRRT: continuous renal replacement therapy; CI: confidence interval; CI: cardiac index; CVP: central venous pressure; DO2I: O2 transport index; HR heart rate; HLOS: hospital length of stay; ICUs: intensive care units; ICULOS: ICU length of stay; Lac: lactate; LVSWI: left ventricular stroke work index; MAP: mean arterial pressure; MPAP: mean pulmonary arterial pressure; NE: norepinephrine; OI: oxygenation index; PAOP: pulmonary artery occlusion pressure; PVRI: pulmonary vascular resistance index; RCTs: randomized controlled trials; RR: relative risk; RAP: right atrial pressure; RVSWI: right ventricular stroke work index; Scr: serum creatinine; SMD: standard mean difference; SVI: stroke volume index; SVRI: systemic vascular resistance index; SvO2: mixed venous oxygen saturation; TP: terlipressin; UO: urine output; VO2I: O2 consumption index; VP: vasopressin.
