# Supplementary material for: Non-catecholamine vasopressors in the treatment of adult patients with septic shock—evidence from meta-analysis and trial sequential analysis of randomized clinical trials
Source: J Intensive Care. 2020 Oct 31;8:83. doi: 10.1186/s40560-020-00500-0 (PMC7603734; doi:10.1186/s40560-020-00500-0)
Supplement: Supplementary file 4 [file 40560_2020_500_MOESM2_ESM.docx]

PICO framework.

| PICO element               Study inclusion criterion |
| --- |
| Patients          Adult patients with septic shock Intervention      Vasopressin、pituitrin、terlipressin、selepressin、angiotensin II Comparison      Norepinephrine Outcomes        The primary endpoints: 28-day mortality; Secondary outcomes included: 90-day, ICU and hospital mortality, ICU length of stay, hospital length of stay, the duration of continuous renal replacement therapy and mechanical ventilation; Serious adverse events: new-onset arrhythmias, life-threatening arrhythmia, mesenteric ischemia, etc; Hemodynamic variables (48h): heart rate, serum creatinine, cardiac index, central venous pressure, O2 transport index, lactate, left ventricular stroke work index, mean arterial pressure, mean pulmonary arterial pressure, oxygenation index, pulmonary artery occlusion pressure, pulmonary vascular resistance index, right atrial pressure, gastric-mucosal arterial carbon dioxide partial pressure difference, PH, right ventricular stroke work index, stroke volume index, systemic vascular resistance index, mixed venous oxygen saturation; urine output, O2 consumption index |

The search strategy and search results

PubMed (n=156), the Cochrane library databases(n=161) and Embase (n=490) 

PubMed:

#1 ((((((((((("Shock, Septic"[Mesh]) OR "Septic Shock"[Title/Abstract]) OR "Toxic Shock"[Title/Abstract]) OR "Toxic Shock Syndrome"[Title/Abstract]) OR "Toxic Shock Syndromes"[Title/Abstract]) OR "Endotoxic Shock"[Title/Abstract]))) OR ((((("Hypovolemic Shock"[Title/Abstract]) OR "Circulatory Collapse"[Title/Abstract]) OR "Circulatory Failure"[Title/Abstract]) OR "Shock"[Title/Abstract]) OR "Shock"[Mesh])) OR ((((((((((((("Sepsis"[Mesh]) OR "Sepsis"[Title/Abstract]) OR "Severe Sepsis"[Title/Abstract]) OR "Sepsis, Severe"[Title/Abstract]) OR "Pyemia"[Title/Abstract]) OR "Pyemias"[Title/Abstract]) OR "Pyohemia"[Title/Abstract]) OR "Pyaemia"[Title/Abstract]) OR "Septicemia"[Title/Abstract]) OR "Septicemias"[Title/Abstract]) OR "Blood Poisoning"[Title/Abstract]) OR "Blood Poisonings"[Title/Abstract]))) OR ((((("Systemic Inflammatory Response Syndrome"[Mesh]) OR "Systemic Inflammatory Response Syndrome"[Title/Abstract]) OR "Sepsis Syndrome"[Title/Abstract]) OR "Sepsis Syndromes"[Title/Abstract]))) OR (((("Bacteremia"[Mesh]) OR "Bacteremia"[Title/Abstract]) OR "Bacteremias"[Title/Abstract]))    369434

#2 ((((((((((((("Vasopressins"[Mesh]) OR "Vasopressins"[Title/Abstract]) OR "beta-Hypophamine"[Title/Abstract]) OR "beta Hypophamine"[Title/Abstract]) OR "Antidiuretic Hormones"[Title/Abstract]) OR "Pitressin"[Title/Abstract]) OR "Vasopressin"[Title/Abstract]))) OR ((((((("Arginine Vasopressin"[Mesh]) OR "Arginine Vasopressin"[Title/Abstract]) OR "Argipressin"[Title/Abstract]) OR "Arg-Vasopressin"[Title/Abstract]) OR "Arg Vasopressin"[Title/Abstract]) OR "Vasopressin, Arginine"[Title/Abstract]))) OR (((((("Pituitary Hormones, Posterior"[Mesh]) OR "Posterior Pituitary Hormones"[Title/Abstract]) OR "Neurohypophyseal Peptides"[Title/Abstract]) OR "Neurohypophyseal Hormones"[Title/Abstract]) OR "Pituitrin"[Title/Abstract]))) OR (((((((((((((("Terlipressin"[Mesh]) OR "Terlipressin"[Title/Abstract]) OR "Glycylpressin"[Title/Abstract]) OR "TGLVP"[Title/Abstract]) OR "Terlypressin"[Title/Abstract]) OR "Triglycylvasopressin"[Title/Abstract]) OR "Glipressin"[Title/Abstract]) OR "Glypressin"[Title/Abstract]) OR "Triglycyl-(8-lysine)vasopressin"[Title/Abstract]) OR "N-(alpha)-glycyl-glycyl-glycyl-8-lysine vasopressin"[Title/Abstract]) OR "Triglycyl Lysine Vasopressin"[Title/Abstract]) OR 2036367[uid]) OR "Remestyp"[Title/Abstract]))) OR "Selepressin*"[Title/Abstract]) OR (((((((((((("Angiotensin II"[Mesh]) OR Angiotensin II[Title/Abstract]) OR "ANG-(1-8)Octapeptide"[Title/Abstract]) OR "Angiotensin-(1-8) Octapeptide"[Title/Abstract]) OR "Valyl(5)-Angiotensin II"[Title/Abstract]) OR "Isoleucyl(5)-Angiotensin II"[Title/Abstract]) OR "Angiotensin*"[Title/Abstract]) OR "Isoleucine(5)-Angiotensin"[Title/Abstract]))))  170122 

#3 ((((((((((("Norepinephrine"[Mesh]) OR "Norepinephrine"[Title/Abstract]) OR "Levonorepinephrine"[Title/Abstract]) OR "Noradrenaline"[Title/Abstract]) OR "Levarterenol"[Title/Abstract]) OR "Levophed"[Title/Abstract]) OR "Levophed Bitartrate"[Title/Abstract]) OR "Norepinephrine Bitartrate"[Title/Abstract]) OR "Noradrenaline Bitartrate"[Title/Abstract]) OR "Norepinephrine Hydrochloride"[Title/Abstract]) OR "Arterenol"[Title/Abstract]) OR "Levonor"         

123227	

#4   ("trial*"[Title/Abstract]) OR ((((random*[Title/Abstract]) OR ("Randomized Controlled Trial" [Publication Type] OR "Randomized Controlled Trials as Topic"[Mesh]))))   1545253

#5 #1 and #2 and #3 and #4   156  (search results)

Cochrane library:

Search Name:	4.30

Last Saved:	30/04/2020 13:42:39

ID	Search

#1	("Shock"):ti,ab,kw OR ("Circulatory Failure"):ti,ab,kw OR ("Failure, Circulatory"):ti,ab,kw OR ("Circulatory Collapse"):ti,ab,kw OR ("Collapse, Circulatory"):ti,ab,kw (Word variations have been searched)

#2	("Hypovolemic Shock"):ti,ab,kw OR ("Shock, Hypovolemic"):ti,ab,kw OR ("Shock, Septic"):ti,ab,kw OR ("Septic Shock"):ti,ab,kw OR ("Shock, Toxic"):ti,ab,kw (Word variations have been searched)

#3	("Toxic Shock"):ti,ab,kw OR ("Toxic Shock Syndrome"):ti,ab,kw OR ("Shock Syndrome, Toxic"):ti,ab,kw OR ("Shock Syndromes, Toxic"):ti,ab,kw OR ("Syndrome, Toxic Shock"):ti,ab,kw (Word variations have been searched)

#4	("Syndromes, Toxic Shock"):ti,ab,kw OR ("Toxic Shock Syndromes"):ti,ab,kw OR ("Shock, Endotoxic"):ti,ab,kw OR ("Endotoxic Shock"):ti,ab,kw OR ("Sepsis"):ti,ab,kw (Word variations have been searched)

#5	("Severe Sepsis"):ti,ab,kw OR ("Sepsis, Severe"):ti,ab,kw OR ("Pyemia"):ti,ab,kw OR ("Pyemias"):ti,ab,kw OR ("Pyohemia"):ti,ab,kw (Word variations have been searched)

#6	("Pyohemias"):ti,ab,kw OR ("Pyaemia"):ti,ab,kw OR ("Pyaemias"):ti,ab,kw OR ("Septicemia"):ti,ab,kw OR ("Septicemias"):ti,ab,kw (Word variations have been searched)

#7	("Poisoning, Blood"):ti,ab,kw OR ("Blood Poisoning"):ti,ab,kw OR ("Blood Poisonings"):ti,ab,kw OR ("Poisonings, Blood"):ti,ab,kw OR ("Systemic Inflammatory Response Syndrome"):ti,ab,kw (Word variations have been searched)

#8	("Inflammatory Response Syndrome, Systemic"):ti,ab,kw OR ("Sepsis Syndrome"):ti,ab,kw OR ("Sepsis Syndromes"):ti,ab,kw OR ("Syndrome, Sepsis"):ti,ab,kw OR ("Syndromes, Sepsis"):ti,ab,kw (Word variations have been searched)

#9	("Bacteremias"):ti,ab,kw OR ("Bacteremia"):ti,ab,kw (Word variations have been searched)

#10	MeSH descriptor: [Shock] explode all trees

#11	MeSH descriptor: [Sepsis] explode all trees

#12	MeSH descriptor: [Systemic Inflammatory Response Syndrome] explode all trees

#13	MeSH descriptor: [Bacteremia] explode all trees

#14	#1 or #2 or #3 or #4 or #5 or #6 or #7 or #8 or #9 or #10 or #11 or #12 or #13   23344

#15	("Angiotensin II"):ti,ab,kw OR ("ANG-(1-8)Octapeptide"):ti,ab,kw OR ("Angiotensin-(1-8) Octapeptide"):ti,ab,kw OR ("Angiotensin II, Val(5)-"):ti,ab,kw OR ("Angiotensin II, Valine(5)-"):ti,ab,kw (Word variations have been searched)

#16	("Valyl(5)-Angiotensin II"):ti,ab,kw OR ("Angiotensin II, Ile(5)-"):ti,ab,kw OR ("Angiotensin II, Isoleucine(5)-"):ti,ab,kw OR ("5-L-Isoleucine Angiotensin II"):ti,ab,kw OR ("5 L Isoleucine Angiotensin II"):ti,ab,kw (Word variations have been searched)

#17	("Angiotensin II, 5-L-Isoleucine"):ti,ab,kw OR ("Isoleucine(5)-Angiotensin"):ti,ab,kw OR ("Isoleucyl(5)-Angiotensin II"):ti,ab,kw OR ("Angiotensin*"):ti,ab,kw OR ("Terlipressin"):ti,ab,kw (Word variations have been searched)

#18	("Glycylpressin"):ti,ab,kw OR ("TGLVP"):ti,ab,kw OR ("Terlypressin"):ti,ab,kw OR ("Triglycyl Lysine Vasopressin"):ti,ab,kw OR ("Triglycylvasopressin"):ti,ab,kw (Word variations have been searched)

#19	("Glipressin"):ti,ab,kw OR ("Glypressin"):ti,ab,kw OR ("Triglycyl-(8-lysine)vasopressin"):ti,ab,kw OR ("Gly-Gly-Gly-8-Lys-vasopressin"):ti,ab,kw OR ("Gly Gly Gly 8 Lys vasopressin"):ti,ab,kw (Word variations have been searched)

#20	("N-(alpha)-glycyl-glycyl-glycyl-8-lysine vasopressin"):ti,ab,kw OR ("Vasopressin, tri-Gly-8-Lys-"):ti,ab,kw OR ("tri-Gly-8-Lys- Vasopressin"):ti,ab,kw OR ("Remestyp"):ti,ab,kw OR ("Vasopressins"):ti,ab,kw (Word variations have been searched)

#21	("beta-Hypophamine"):ti,ab,kw OR ("beta Hypophamine"):ti,ab,kw OR ("Antidiuretic Hormones"):ti,ab,kw OR ("Pitressin"):ti,ab,kw OR ("Vasopressin (USP)"):ti,ab,kw (Word variations have been searched)

#22	("Vasopressin"):ti,ab,kw OR ("Arginine Vasopressin"):ti,ab,kw OR ("Argipressin"):ti,ab,kw OR ("Arg-Vasopressin"):ti,ab,kw OR ("Arg Vasopressin"):ti,ab,kw (Word variations have been searched)

#23	("Vasopressin, Arginine"):ti,ab,kw OR ("Argipressin Tannate"):ti,ab,kw OR ("Pituitary Hormones, Posterior"):ti,ab,kw OR ("Posterior Pituitary Hormones"):ti,ab,kw OR ("Neurohypophyseal Peptides"):ti,ab,kw (Word variations have been searched)

#24	("Neurohypophyseal Hormones"):ti,ab,kw OR ("Pituitrin"):ti,ab,kw OR ("Selepressin*"):ti,ab,kw (Word variations have been searched)

#25	MeSH descriptor: [Pituitary Hormones, Posterior] explode all trees

#26	MeSH descriptor: [Angiotensin II] explode all trees

#27	MeSH descriptor: [Terlipressin] explode all trees

#28	MeSH descriptor: [Vasopressins] explode all trees

#29	MeSH descriptor: [Arginine Vasopressin] explode all trees

#30	#15 or #16 or #17 or #18 or #19 or #20 or #21 or #22 or #23 or #24 or #25 or #26 or #27 or #28 or #29  18692

#31	("Norepinephrine"):ti,ab,kw OR ("Levonorepinephrine"):ti,ab,kw OR ("Noradrenaline"):ti,ab,kw OR ("Levarterenol"):ti,ab,kw OR ("Levophed"):ti,ab,kw (Word variations have been searched)

#32	MeSH descriptor: [Norepinephrine] explode all trees

#33	("Levophed Bitartrate"):ti,ab,kw OR ("Noradrénaline tartrate renaudin"):ti,ab,kw OR ("Norepinephrine Bitartrate"):ti,ab,kw OR ("Noradrenaline Bitartrate"):ti,ab,kw OR ("Norepinephrine Hydrochloride"):ti,ab,kw

#34	("Norepinephrine Hydrochloride, (+)-Isomer"):ti,ab,kw OR ("Norepinephrine Hydrochloride, (+,-)-Isomer"):ti,ab,kw OR ("Norepinephrine l-Tartrate (1:1), (+,-)-Isomer"):ti,ab,kw OR ("Norepinephrine l-Tartrate (1:1), Monohydrate"):ti,ab,kw OR ("Norepinephrine l-Tartrate (1:1), Monohydrate, (+)-Isomer"):ti,ab,kw

#35	("Norepinephrine l-Tartrate, (+)-Isomer"):ti,ab,kw OR ("Norepinephrine, (+)-Isomer"):ti,ab,kw OR ("Norepinephrine, (+,-)-Isomer"):ti,ab,kw OR ("Arterenol"):ti,ab,kw OR ("Levonor"):ti,ab,kw

#36	#31 or #32 or #33 or #34 or #35  7978

#37	("trial*"):ti,ab,kw OR ("random*"):ti,ab,kw OR ("randomized controlled trial"):ti,ab,kw (Word variations have been searched)

#38	MeSH descriptor: [Randomized Controlled Trial] explode all trees

#39	#37 or #38  1071438

#40	#14 and #30 and #36 and #39  161  (search results)

Embase Session Results (30 Apr 2020) （search results: 490）

Embase

Session Results

.......................................................

No.        Query Results                          Results        Date       

#136.#48 AND #104 AND #130 AND #135              490     30 Apr 2020

#135.#131 OR #132 OR #133 OR #134                2,507,429  30 Apr 2020

#134.'randomized controlled trial (topic)'/exp            177,426  30 Apr 2020

#133.'randomized controlled trial'/exp                598,996  30 Apr 2020

#132.'random*':ab,ti                               1,528,914  30 Apr 2020

#131.'trial*':ab,ti                                  1,455,937  30 Apr 2020

#130.#105 OR #106 OR #107 OR #108 OR #109 OR #110 OR        175,048  30 Apr 2020

     #111 OR #112 OR #113 OR #114 OR #115 OR #116 OR 

     #117 OR #118 OR #119 OR #120 OR #121 OR #122 OR 

     #123 OR #124 OR #125 OR #126 OR #127 OR #128 OR 

     #129

#129.'noradrenalin'/exp                           143,388  30 Apr 2020

#128.'levonor':ab,ti                                  13  30 Apr 2020

#127.'arterenol':ab,ti                                699  30 Apr 2020

#126.'norepinephrine, (+,-)-isomer':ab,ti                  30 Apr 2020

#125.'norepinephrine, (+)-isomer':ab,ti                      30 Apr 2020

#124.'norepinephrine l-tartrate, (+)-isomer':ab,ti               30 Apr 2020

#123.'norepinephrine l-tartrate (1:2)':ab,ti                    30 Apr 2020

#122.'norepinephrine l-tartrate (1:1), monohydrate,            30 Apr 2020

     (+)-isomer':ab,ti

#121.'norepinephrine l-tartrate (1:1),                        30 Apr 2020

     monohydrate':ab,ti

#120.'norepinephrine l-tartrate (1:1),                        30 Apr 2020

     (+,-)-isomer':ab,ti

#119.'norepinephrine hydrochloride,                        30 Apr 2020

     (+,-)-isomer':ab,ti

#118.'norepinephrine hydrochloride, (+)-isomer':ab,ti           30 Apr 2020

#117.'norepinephrine hydrochloride':ab,ti                 23  30 Apr 2020

#116.'norepinephrin d-tartrate (1:1)':ab,ti                     30 Apr 2020

#115.'norepinephrine d-tartrate (1:1)':ab,ti                     30 Apr 2020

#114.'noradrenaline bitartrate':ab,ti                      29  30 Apr 2020

#113.'norepinephrine l-tartrate (1:1)':ab,ti                     30 Apr 2020

#112.'norepinephrine bitartrate':ab,ti                     55  30 Apr 2020

#111.'noradrénaline tartrate renaudin':ab,ti                    30 Apr 2020

#110.'levophed bitartrate':ab,ti                           6  30 Apr 2020

#109.'levophed':ab,ti                                 129  30 Apr 2020

#108.'levarterenol':ab,ti                               282  30 Apr 2020

#107.'noradrenaline':ab,ti                           47,656  30 Apr 2020

#106.'levonorepinephrine':ab,ti                               30 Apr 2020

#105.'norepinephrine':ab,ti                           70,080  30 Apr 2020

#104.#49 OR #50 OR #51 OR #52 OR #53 OR #54 OR #55 OR       211,795  30 Apr 2020

     #56 OR #57 OR #58 OR #59 OR #60 OR #61 OR #62 OR 

     #63 OR #64 OR #65 OR #66 OR #67 OR #68 OR #69 OR 

     #70 OR #71 OR #72 OR #73 OR #74 OR #75 OR #76 OR 

     #77 OR #78 OR #79 OR #80 OR #81 OR #82 OR #83 OR 

     #84 OR #85 OR #86 OR #87 OR #88 OR #89 OR #90 OR 

     #91 OR #92 OR #93 OR #94 OR #95 OR #96 OR #97 OR 

     #98 OR #99 OR #100 OR #101 OR #102 OR #103

#103.'angiotensin ii'/exp                              30,628  30 Apr 2020

#102.'angiotensin'/exp                               38,500  30 Apr 2020

#101.'terlipressin'/exp                                3,017  30 Apr 2020

#100.'vasopressin'/exp                               38,949  30 Apr 2020

#99. 'argipressin'/exp                                12,874  30 Apr 2020

#98. 'pituitrin'/exp                                    786  30 Apr 2020

#97. 'selepressin'/exp                                  50  30 Apr 2020

#96. 'selepressin*':ab,ti                                27  30 Apr 2020

#95. 'pituitrin':ab,ti                                   731  30 Apr 2020

#94. 'neurohypophyseal hormones':ab,ti                  405  30 Apr 2020

#93. 'neurohypophyseal peptides':ab,ti                    152  30 Apr 2020

#92. 'posterior pituitary hormones':ab,ti                   236  30 Apr 2020

#91. 'pituitary hormones, posterior':ab,ti                   1  30 Apr 2020

#90. 'argipressin tannate':ab,ti                              30 Apr 2020

#89. 'vasopressin, arginine':ab,ti                        57  30 Apr 2020

#88. 'arg vasopressin':ab,ti                            167  30 Apr 2020

#87. 'arg-vasopressin':ab,ti                            167  30 Apr 2020

#86. 'argipressin':ab,ti                                38  30 Apr 2020

#85. 'arginine vasopressin':ab,ti                      11,943  30 Apr 2020

#84. 'vasopressin':ab,ti                             40,435  30 Apr 2020

#83. 'vasopressin (usp)':ab,ti                               30 Apr 2020

#82. 'pitressin':ab,ti                                 1,086  30 Apr 2020

#81. 'antidiuretic hormones':ab,ti                       88  30 Apr 2020

#80. 'beta hypophamine':ab,ti                           3  30 Apr 2020

#79. 'beta-hypophamine':ab,ti                           3  30 Apr 2020

#78. 'vasopressins':ab,ti                               113  30 Apr 2020

#77. 'remestyp':ab,ti                                  11  30 Apr 2020

#76. 'tri-gly-8-lys- vasopressin':ab,ti                          30 Apr 2020

#75. 'vasopressin, tri-gly-8-lys-':ab,ti                         30 Apr 2020

#74. 'n-(alpha)-glycyl-glycyl-glycyl-8-lysine                  30 Apr 2020

vasopressin':ab,ti

#73. 'gly gly gly 8 lys vasopressin':ab,ti                       30 Apr 2020

#72. 'gly-gly-gly-8-lys-vasopressin':ab,ti                      30 Apr 2020

#71. 'triglycyl-(8-lysine)vasopressin':ab,ti                  9  30 Apr 2020

#70. 'glypressin':ab,ti                                 102  30 Apr 2020

#69. 'glipressin':ab,ti                                   3  30 Apr 2020

#68. 'triglycylvasopressin':ab,ti                          14  30 Apr 2020

#67. 'triglycyl lysine vasopressin':ab,ti                    41  30 Apr 2020

#66. 'terlypressin':ab,ti                                 3  30 Apr 2020

#65. 'tglvp':ab,ti                                      26  30 Apr 2020

#64. 'glycylpressin':ab,ti                                4  30 Apr 2020

#63. 'terlipressin':ab,ti                                1,341  30 Apr 2020

#62. 'angiotensin*':ab,ti                              139,640  30 Apr 2020

#61. 'isoleucyl(5)-angiotensin ii':ab,ti                      1  30 Apr 2020

#60. 'isoleucine(5)-angiotensin':ab,ti                       3  30 Apr 2020

#59. 'angiotensin ii, 5-l-isoleucine':ab,ti                        30 Apr 2020

#58. '5 l isoleucine angiotensin ii':ab,ti                         30 Apr 2020

#57. '5-l-isoleucine angiotensin ii':ab,ti                         30 Apr 2020

#56. 'angiotensin ii, isoleucine(5)-':ab,ti                        30 Apr 2020

#55. 'angiotensin ii, ile(5)-':ab,ti                           1  30 Apr 2020

#54. 'valyl(5)-angiotensin ii':ab,ti                          3  30 Apr 2020

#53. 'angiotensin ii, valine(5)-':ab,ti                        2  30 Apr 2020

#52. 'angiotensin ii, val(5)-':ab,ti                             30 Apr 2020

#51. 'angiotensin-(1-8) octapeptide':ab,ti                   16  30 Apr 2020

#50. 'ang-(1-8)octapeptide':ab,ti                           4  30 Apr 2020

#49. 'angiotensin ii':ab,ti                              63,276  30 Apr 2020

#48. #1 OR #2 OR #3 OR #4 OR #5 OR #6 OR #7 OR #8 OR        560,767  30 Apr 2020

     #9 OR #10 OR #11 OR #12 OR #13 OR #14 OR #15 OR 

     #16 OR #17 OR #18 OR #19 OR #20 OR #21 OR #22 OR 

     #23 OR #24 OR #25 OR #26 OR #27 OR #28 OR #29 OR 

     #30 OR #31 OR #32 OR #33 OR #34 OR #35 OR #36 OR 

     #37 OR #38 OR #39 OR #40 OR #41 OR #42 OR #43 OR 

     #44 OR #45 OR #46 OR #47

#47. 'bacteremia'/exp                                  51,243  30 Apr 2020

#46. 'systemic inflammatory response syndrome'/exp        280,290  30 Apr 2020

#45. 'sepsis'/exp                                    273,344  30 Apr 2020

#44. 'septic shock'/exp                                 52,969  30 Apr 2020

#43. 'shock'/exp                                    145,306  30 Apr 2020

#42. 'bacteremias':ab,ti                                1,546  30 Apr 2020

#41. 'bacteremia':ab,ti                                 31,545  30 Apr 2020

#40. 'syndromes, sepsis':ab,ti                              8  30 Apr 2020

#39. 'syndrome, sepsis':ab,ti                             358  30 Apr 2020

#38. 'sepsis syndromes':ab,ti                             113  30 Apr 2020

#37. 'sepsis syndrome':ab,ti                             1,058  30 Apr 2020

#36. 'inflammatory response syndrome, systemic':ab,ti         2  30 Apr 2020

#35. 'systemic inflammatory response syndrome':ab,ti       7,012  30 Apr 2020

#34. 'poisonings, blood':ab,ti                              1  30 Apr 2020

#33. 'blood poisonings':ab,ti                               1  30 Apr 2020

#32. 'blood poisoning':ab,ti                               34  30 Apr 2020

#31. 'poisoning, blood':ab,ti                              80  30 Apr 2020

#30. 'septicemias':ab,ti                                  452  30 Apr 2020

#29. 'septicemia':ab,ti                                 17,112  30 Apr 2020

#28. 'pyaemias':ab,ti                                     2  30 Apr 2020

#27. 'pyaemia':ab,ti                                    145  30 Apr 2020

#26. 'pyohemias':ab,ti                                       30 Apr 2020

#25. 'pyohemia':ab,ti                                   6  30 Apr 2020

#24. 'pyemias':ab,ti                                    4  30 Apr 2020

#23. 'pyemia':ab,ti                                    94  30 Apr 2020

#22. 'sepsis, severe':ab,ti                              952  30 Apr 2020

#21. 'severe sepsis':ab,ti                              14,386  30 Apr 2020

#20. 'sepsis':ab,ti                                   149,266  30 Apr 2020

#19. 'endotoxic shock':ab,ti                            2,512  30 Apr 2020

#18. 'shock, endotoxic':ab,ti                             18  30 Apr 2020

#17. 'toxic shock syndromes':ab,ti                        44  30 Apr 2020

#16. 'syndromes, toxic shock':ab,ti                        1  30 Apr 2020

#15. 'syndrome, toxic shock':ab,ti                         12  30 Apr 2020

#14. 'shock syndromes, toxic':ab,ti                            30 Apr 2020

#13. 'shock syndrome, toxic':ab,ti                          6  30 Apr 2020

#12. 'toxic shock syndrome':ab,ti                        4,327  30 Apr 2020

#11. 'toxic shock':ab,ti                                 5,342  30 Apr 2020

#10. 'shock, toxic':ab,ti                                  18  30 Apr 2020

#9.  'septic shock':ab,ti                               34,868  30 Apr 2020

#8.  'shock, septic':ab,ti                                158  30 Apr 2020

#7.  'shock, hypovolemic':ab,ti                           26  30 Apr 2020

#6.  'hypovolemic shock':ab,ti                           3,395  30 Apr 2020

#5.  'collapse, circulatory':ab,ti                           3  30 Apr 2020

#4.  'circulatory collapse':ab,ti                          1,996  30 Apr 2020

#3.  'failure, circulatory':ab,ti                            56  30 Apr 2020

#2.  'circulatory failure':ab,ti                           3,973  30 Apr 2020

#1.  'shock':ab,ti                                   247,273  30 Apr 2020
